# Impermeability and Durability of Self-Compacting Concrete Prepared with Aeolian Sand and Recycled Coarse Aggregate

**DOI:** 10.3390/ma16237279

**Published:** 2023-11-22

**Authors:** Shiqi Zheng, Qing Liu, Fengxia Han, Shan Liu, Guoxing Zhang, Jiayan Zhu

**Affiliations:** 1 College of Civil Engineering and Architecture, Xinjiang University, Urumqi 830017, China; 107552101534@stu.xju.edu.cn (S.Z.); zhangguoxing1@stu.xju.edu.cn (G.Z.); 107552104159@stu.xju.edu.cn (J.Z.); 2 Key Laboratory of Building Structure and Seismic Resistance of Xinjiang, Urumqi 830017, China; fxhan@xju.edu.cn (F.H.); liushan@xju.edu.cn (S.L.)

**Keywords:** self-compacting concrete, aeolian sand, recycled coarse aggregate, sulfate attack, impermeability

## Abstract

Self-compacting concrete has seen extensive application in both engineering and construction. In order to save building resources, aeolian sand—recycled coarse aggregate self-compacting concrete (ARSCC) is created by partially substituting recycled coarse aggregates (RCA) and aeolian sand (AS) for natural coarse aggregates. For ten groups with different mechanical and durable properties, this study examined the effects of sulfate erosion, chloride penetration resistance, and related impermeability, as well as AS replacement ratios of 20%, 40%, and 60% and RCA replacement ratios of 25%, 50%, and 75% in ARSCC and a control group (A0-R0). According to the study’s findings, after sulfate attack, the highest relative dynamic elastic modulus and corrosion resistance factor were obtained with the 20% AS replacement ratio and 50% RCA replacement ratio (A20-R50). The highest impermeability grade and lowest electric flux were obtained with the 20% AS replacement ratio and 25% RCA replacement ratio (A20-R25). X-ray diffraction (XRD) and mercury intrusion porosimetry (MIP) revealed that the addition of aeolian sand and recycled coarse aggregates improved the pore structure of the SCC and increased the densification of the self-compacting concrete, particularly following sulfate attack. This study highlights the importance of recycled aggregates and aeolian sand in engineering applications and the sustainable growth of the concrete industry, both of which support resource conservation and environmental protection.

## 1. Introduction

The quality, appropriate use, and environmental impact of recycled materials present a difficult situation in the construction business today. In order to produce high-quality concrete, which can have aggregate compositions of up to three-fourths of the volume of the concrete, the construction industry needs a vast amount of natural resources due to increased urbanization [1]. Therefore, alternative materials from construction and demolition (C&D) waste and less-used natural resources can be used in the production of concrete to address the issue of the excessive use of aggregates (fine and coarse). This reduces land waste, lowers the cost of concrete, and conserves valuable natural resources. Currently, employing aggregate recycled from concrete and construction is the most promising choice out of a variety of options. In this study, less-utilized natural resources such as aeolian sand (AS) from deserts were selected.

In an effort to increase the longevity of concrete constructions, self-compacting concrete (SCC) was introduced in 1988 [2]. Subsequently, SCC has had significant growth and has had a significant impact on civil engineering, being applied to a wide range of construction projects worldwide, including office buildings, tunnels in Japan, highway bridges in Sweden, and useful structures in China, the United States, the Netherlands, and Thailand [3,4,5]. Recycled coarse aggregate self-compacting concrete (RCASCC), which combines recycled coarse aggregate (RCA) with SCC, has been the subject of extensive research due to the growing awareness of human impact on the environment. The most common replacement ratios are 25%, 50%, and 75% [6,7,8,9,10,11,12]. Therefore, the use of RCASCC is both technically feasible and justified; nevertheless, care must be taken to ensure that the performance characteristics of this type of concrete are adequate to meet the requirements of each individual situation [13].

Globally available aeolian sand can be converted into a green resource through appropriate processing and use [14,15]. Due to the scarcity of natural resources in recent years, a large number of researchers have examined the use of aeolian sand (AS) in concrete globally. Their research primarily focuses on AS as a raw material for concrete, with the expectation that it will eventually serve as the primary fine aggregate in concrete and construction [16,17,18,19]. There are two primary results on AS replacement: (1) there exists an ideal ratio for AS replacement [20,21,22], and (2) the use of AS alone has adverse impacts on the characteristics of concrete and construction [15,17,23]. Consequently, research on local deserts is required, and the use of AS in concrete is both technically feasible and warranted.

Numerous studies have been conducted on the mechanical and durability characteristics of aeolian sand concrete, RCASCC, and recycled aggregate concrete. Trends in the durability characteristics of concrete made with RCA and AS [24] have been documented in the literature. These characteristics include water permeability [25,26], resistance to chloride penetration [27,28], sulfate attack [29,30], and freeze–thaw resistance [31,32,33]. In conclusion, SCC allows for the addition of AS and RCA.

Thus, this study’s goal is to lower building costs and reinforce the idea of sustainability by employing recycled coarse aggregates and fine materials such as AS. Furthermore, it examines and emphasizes how replacing these materials affects water permeability and the long-term effectiveness of ARSCC in sulfate settings.

## 2. Materials and Methods

### 2.1. Materials

#### 2.1.1. Binder, Admixtures, and Water

Fly ash from the Xinjiang Zhongtian Yintai firm and PO42.5 Portland cement, which complies with the standard, were utilized as binders in all concrete combinations during the production process. The specific gravity and specific surface area of Portland cement are 354 m^2^/kg. The cubic concrete specimens prepared for this study had a strength rating of C30. Table 1 lists the characteristic features of Portland cement, and Table 2 lists the fly ash’s performance.

The fresh concrete was made sufficiently workable, and the water–binder ratio was lowered by using a water reducer with a water reduction rate of roughly 25–30%. The water utilized in this study is from Urumqi, where water is typically sourced from to make concrete.

#### 2.1.2. Fine Aggregate

**Figure 1:**
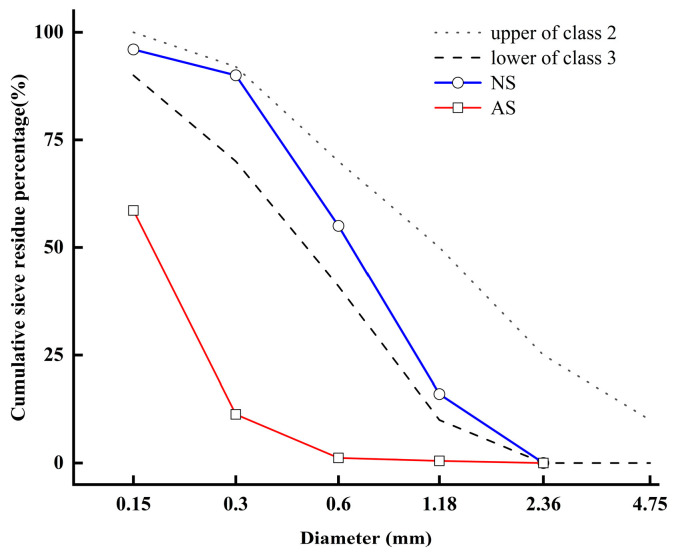
Particle size distributions of the sand.

To make ARSCC, aeolian sand was extracted from the Gurbantungut Desert in northern Xinjiang. The ARSCC’s performance is greatly impacted by the characteristics of aeolian sand. This study’s fine aggregate’s fundamental physical characteristics are displayed in Table 3. Figure 1 shows the fine aggregate particle size distributions obtained via sieve analysis.

#### 2.1.3. Coarse Aggregate

**Figure 2:**
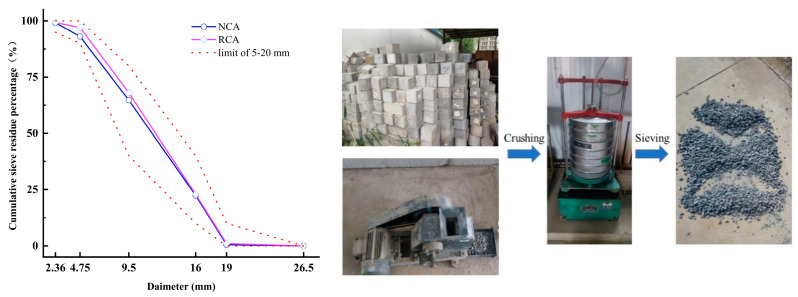
Productive process and particle size distributions of the coarse aggregate.

Recycled concrete aggregates were derived from Urumqi’s concrete waste. The research generally agrees that there is less mortar adhered to coarse recycled aggregate than fine recycled aggregate [34,35]. This results in the RCA having a greater quality than the fine fraction of recycled aggregate, and in this sense, it makes sense to improve the quality of a material that is relatively better than a lower-quality material [36]. According to this study, the proportion of aggregate retained between 2.36 and 19 mm sieves is known as the RCA. Table 4 displays the fundamental physical characteristics of the coarse aggregate employed in this investigation. Figure 2 shows the coarse aggregate particle size distributions obtained via sieve analysis.

### 2.2. Sample Preparation

Table 5 displays the proportions of the mixture. While mass substitution was chosen for engineering applications and ease of calculation because the density difference between NS and AS is only 54.9 kg/m^3^, volume substitution was chosen to preserve the stability of the concrete’s constituent parts after substitution and the substitution rate. The AS replacement ratios of 0%, 20%, 40%, and 60% by mass are represented by A0, A20, A40, and S60; the RCA replacement ratios of 0%, 25%, 50%, and 75% by volume are represented by R0, R25, R50, and R75. Water-to-binder (W/B) ratio was 0.33. Prior to molding, the experimental mixes including the different AS and RCA replacement ratios underwent a workability test, as indicated in Table 6. The specimens were poured, left for 24 h, and then cured for 28 days at 20 °C and 95% relative humidity under conventional curing conditions before being demold. To obtain the average value, each test sample was measured three times per group.

### 2.3. Workability Tests

**Figure 3:**
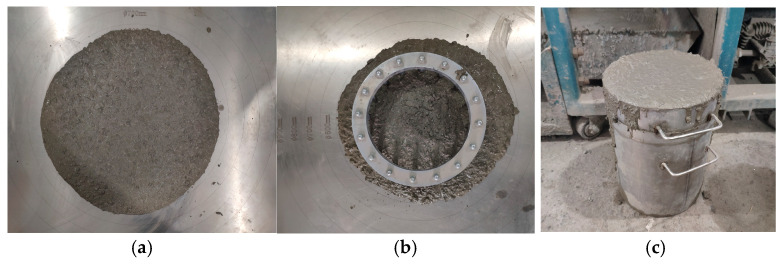
Workability tests of fresh ARSCC. (**a**) Slump flow. (**b**) J-ring. (**c**) Segregation resistance.

As seen in Figure 3, the workability test of fresh ARSCC was carried out in compliance with specifications. Three common behaviors were shown on the workability test: segregation, passing, and filling abilities. In slump flow experiments, the viscosity and flowability of the new mix were evaluated by measuring the slump flow diameter. The segregation of fresh ARSCC was assessed using segregation resistance, and the passage ability was assessed using the J-ring test.

### 2.4. Compressive Strength Tests

After 28 days of curing, compressive strength tests were performed on all ARSCC mixes in compliance with the standard. Cube specimens measuring 150 mm × 150 mm × 150 mm and 100 mm × 100 mm × 100 mm were prepared for the tests.

### 2.5. Resistance Water and Chloride Penetration Tests

Hard ARSCC’s resistance to chloride penetration was determined with coulomb electric flux tests, and its resistance to water penetration was determined with progressive pressure loading tests using standard procedures. Furthermore, the dimensions of the test components are as follows: for water penetration testing, the round table body was Ø 175 mm × Ø 185 mm × 150 mm, and for electric flux tests, the cylinder measured Ø 100 mm × 50 mm.

#### 2.5.1. Gradual Pressure Loading Tests

For four of the six examples in each group, the maximum seepage pressure multiplied by 10 yielded the grade of seepage resistance of the concrete in the absence of seepage. The formula for calculating the seepage resistance grade of concrete is P = 10H − 1, where P represents the impermeability grade and H denotes the seepage pressure (MPa) at which three out of the six specimens experience seepage.

#### 2.5.2. Electric Flux Tests

**Figure 4:**
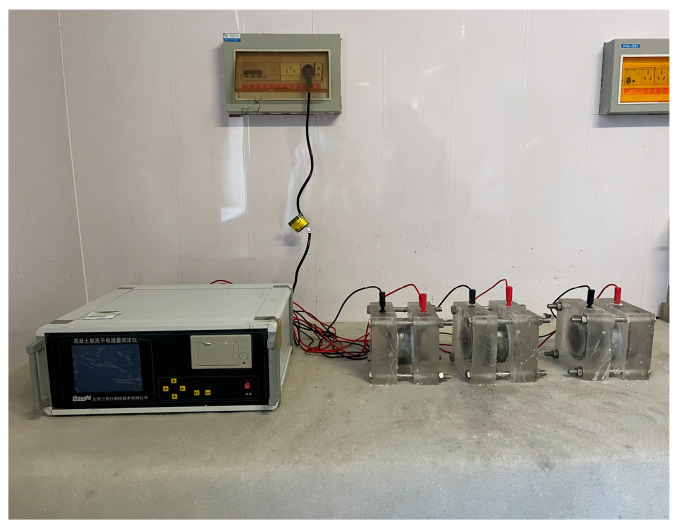
Electric flux test.

NaCl solution with a mass concentration of 3% and NaOH solution with a molar concentration of 0.3 mol/L were simultaneously injected into both sides of the test tank to provide access to 60 V direct current after the specimen was placed into the specimen tank to verify the device’s sealing. The initial current reading, I0, was recorded with the positive electrode on the NaOH side and the negative electrode on the NaCl side. As seen in Figure 4, the current value was collected both during and after the test, and it remained active for six hours.

### 2.6. Resistance to Sulfate Attack Tests

**Figure 5:**
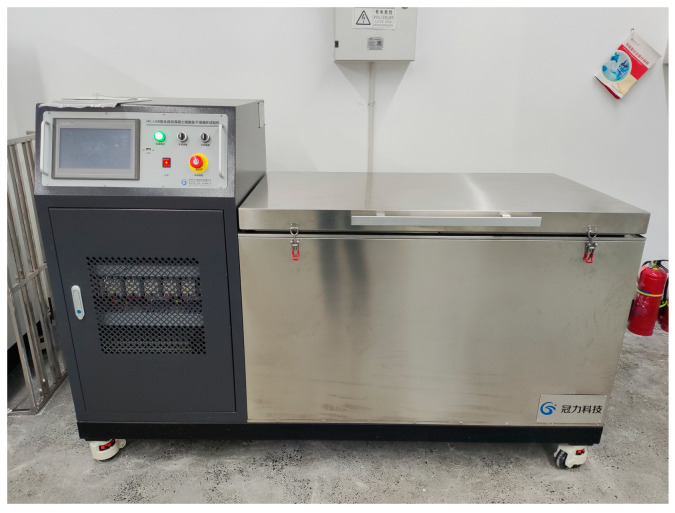
HC-LSB concrete sulfate dry–wet cycle testing machine.

Hard ARSCC tests were used to determine how resistant the concrete was to sulfate attacks using established methods. The specimens in this study were totally submerged in a 5% Na_2_SO_4_ solution for 15 h, followed by 6 h of drying as part of a 22 h drying–wetting process using HC-LSB concrete sulfate dry–wet cycle testing equipment (Figure 5). For 120 days, there were cycles of drying and soaking. The test pieces were designed as cube specimens (100 mm × 100 mm × 100 mm) for compressive strength and prism specimens (100 mm × 100 mm × 400 mm) for mass and elasticity variation.

#### 2.6.1. Mass Variation

With an accuracy of 0.1 g, the mass variations in the specimens under various dry–wet cycles were measured using an electronic balance. After the specimens were dried for 48 h at 45 °C in an oven, the mass was measured. The formula D_mi_ = (mi − m0) 100%/m0, where m0 is the starting mass and mi is the mass after N of the dry and wet cycle, can be used to calculate the mass change D_m_.

#### 2.6.2. Relative Dynamic Modulus of Elasticity

Using a nonmetallic ultrasonic testing analyzer, the concrete’s dynamic modulus of elasticity was determined. The sound velocity of the initial peak value of an ultrasonic wave traveling through the specimens was measured with the instrument, and the sound speed was translated into the concrete’s dynamic elastic modulus. Equations (1) and (2) provide an illustration of the precise formula:

(1) $$P_{i}=\frac{f_{ni}^{2}}{f_{0i}^{2}}$$

(2) $$P=\frac{1}{3}\sum_{1=1}^{3}P_{i}$$

where P_i_ is the relative dynamic elastic modulus of the i th concrete sample after N dry and wet cycles; $f_{ni}$ is the transverse fundamental frequency of the i th concrete test block after N dry and wet cycles; $f_{0i}$ is the initial value of transverse fundamental frequency of the i th concrete sample after N dry and wet cycles; and P is the relative dynamic elastic modulus of a group of concrete sample after N dry and wet cycles.

#### 2.6.3. Corrosion Resistance Factor

The ratio of the compressive strength of concrete specimens subjected to sulfate dry–wet cycle erosion to the compressive strength of the concrete specimens subjected to ordinary curing is known as the corrosion resistance factor [37]. Equation (3) provides an illustration of the exact formula:

(3) $$K_{f}=\frac{f_{cn}}{f_{c0}}\times 100\%$$

where $K_{f}$ is the corrosion resistance factor (%); $f_{cn}$ is the compressive strength of a group of concrete specimens after N wet and dry sulfate cycles; and $f_{c0}$ is the compressive strength of a group of concrete specimens under standard curing.

### 2.7. X-ray Diffraction (XRD)

An X-ray diffractometer (Bruker D8 advance) was utilized to assess the substances’ composition and content. Samples of cement mortar without gravel were chosen from the same group of sample fragments and crushed, mixed evenly, and filtered using a 300 mesh screen after the compressive strength test was finished. In the end, 1 g of each sample group was extracted for the X-ray diffraction (XRD) test. Following the test, all of the XRD samples were extracted from the pieces and pulverized in an agate mortar. The data files that were collected were then processed using MDI Jade, and graphs were created. XRD was carried out in the 2θ range of 10–75° at a scanning speed of 2°/min in the 2θ range of.

### 2.8. Mercury Intrusion Porosimetry (MIP)

An AutoPore Iv 9510 from American Micromeritics was utilized to perform mercury intrusion porosimetry (MIP) testing, which measures the changes in pore structure characteristics caused by sulfate assault. The pressure range tested was 0.2 MPa to 415 MPa. By chopping the concrete into roughly 1 cm^3^ cubes and drying it in the same conditions as the XRD experiments, the concrete samples for the MIP were taken from the surface of concrete cylinder specimens.

## 3. Results and Discussion

### 3.1. Workability and Compressive Strength

Table 7 shows the workability of fresh concrete in terms of segregation, filling ability, and passing ability. The figure illustrates the ARSCC’s slump flow, segregation resistance, and J-ring. Given the disparity in the replacement ratios between A0-R0 and A60-R75, this suggests that the AS and RCA replacement ratios negatively impact the flowability and passing ability of fresh ARSCC. The workability of fresh ARSCC is not significantly affected by the replacement ratios of 25% and 50%, nor by the replacement ratio of 20% AS. In terms of flowability, A60-R75’s slump flow is 554 mm, which is 102 mm less than that of the comparison group A0-R0. In addition, the J-ring shrank from 12 mm to 1 mm, exhibiting a clear propensity toward blockage. Furthermore, all ARSCC segregation resistance values are below 12%, suggesting a higher degree of segregation.

**Figure 6:**
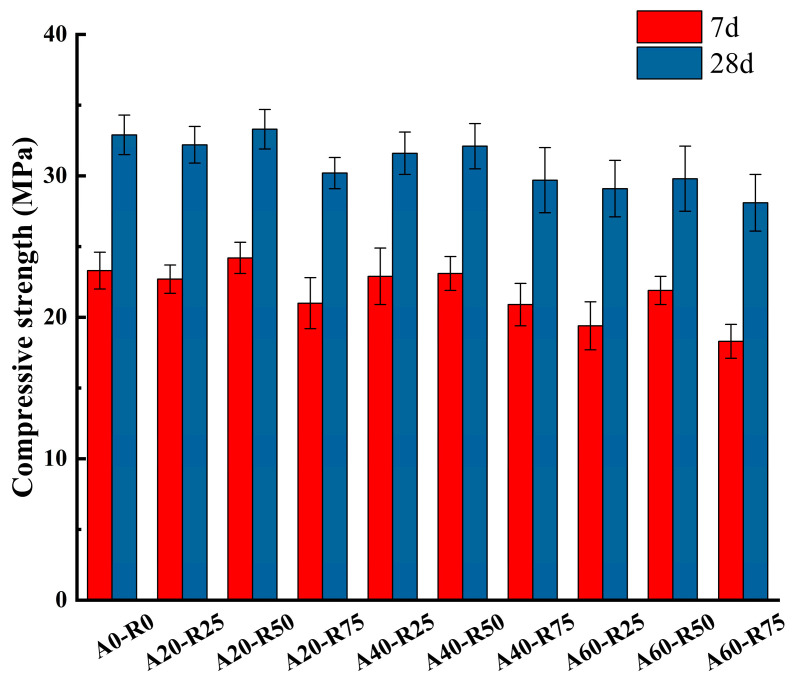
Compressive strength of ARSCC with different replacement ratios.

Figure 6 illustrates the impact the impact of the SA and RCA replacement ratios on the compressive strength of ARSCC at 7 and 28 days of curing. In the figure, it can be observed that when the AS is fixed, the compressive strength increases initially before gradually decreasing as the RCA replacement ratio increases. The inflection point occurs at the 50% replacement ratio. Conversely, when RCA is fixed, the compressive strength gradually decreases as the AS replacement ratio increases. Comparing the compressive strength to the control group, A0-R0, there was a maximum increase of 0.4 MPa and a minimum decrease of 4.8 MPa; the highest value was 33.3 MPa for A20-R50, and the minimum value was 28.1 MPa for A60-R75. This suggests that replacing AS and RCA does not significantly affect the compressive strength of hard ARSCC, given that replacing SA with an appropriate ratio and NCA with a similar RCA content optimizes the gradation of concrete aggregates and increases compactness.

### 3.2. Impermeability Grade and Electric Flux

Table 8 displays the electric flux and maximum water pressure test results. It appears that when the replacement ratios of AS and RCA increase, the trends of the impermeability grade and electric flux decrease. In comparison to the control group (A0-R0), A20-R25 and A20-R50 exhibit superior impermeability and resistance to chloride penetration. In fact, their electric flux and impermeability grade increased by one grade and decreased by 9.83 C, respectively. However, mistakes in the device’s measurement could be the reason for the anomalous electric flux of A60-R25. The compactness of ARSCC is somewhat improved by the replacement of AS and RCA. In order to increase the compactness of ARSCC, AS can be used to fill the smaller pores at a low replacement ratio and give the cement greater surface area for hydration. This demonstrates that ARSCC produced with an appropriate replacement ratio can perform on par with or even better than the control concrete.

**Figure 7:**
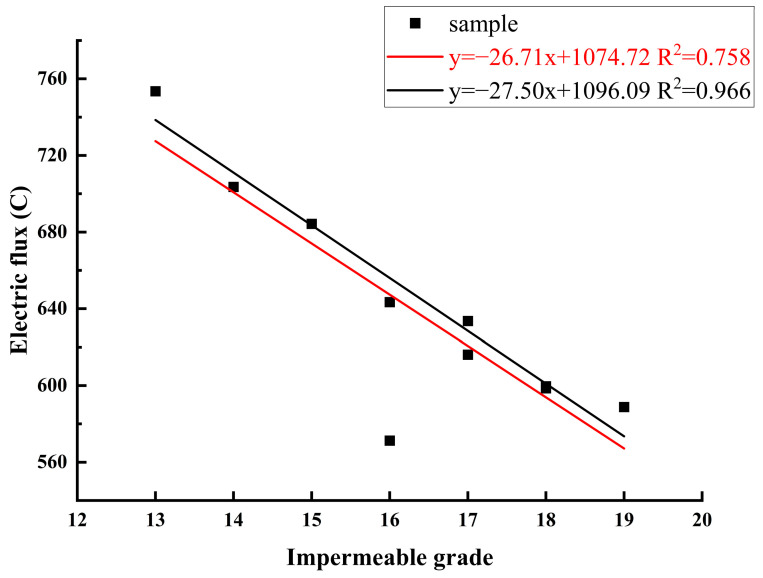
The relationship of impermeability grade and electric flux.

Figure 7 illustrates the relationship between the electric flux and the impermeability grade. After linear fitting, it was discovered that there is a distinct linear association (R^2^ = 0.758); if the A60-R25 sample is removed, R^2^ = 0.996 is achieved. The 6 h electric flux of ARSCC exhibits a declining trend as the impermeability grade rises. As a result, water finds it more difficult to pass through ARSCC at certain pressures, which is also indicative of the material’s increasing compactness, decreasing internal pore space, and steadily declining total permeability. Chloride ions also find it challenging to pass through ARSCC. As the impermeability grade rises, the electric flux tends to decrease. The ideal relationship between concrete’s electric flux and impermeability grade would be a strong linear one. However, because ARSCC is a non-homogeneous material, interference from multiple factors might affect its own state. Additionally, there will be varying degrees of impact on the concrete’s exterior morphology and interior structure in the specimen used for molding, curing, vibration, grinding, and other tests. As a result, ARSCC’s attributes cannot be fully represented in a linear relationship during the fitting process.

### 3.3. Durability

#### 3.3.1. Visual Examination

**Figure 8:**
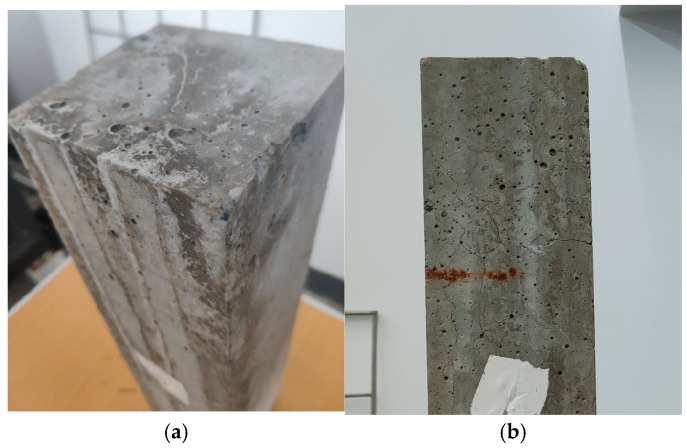
Visual inspection of ARSCC after sulfate attack: (**a**) 60 cycles, (**b**) 120 cycles.

Figure 8 illustrates the visual examination of ARSCC prism specimens subjected to 0, 60, and 120 days of sulfate dry–wet cycle erosion. The ARSCCs exhibited negligible surface degradation overall.

The specimens show varying degrees of damage following varying periods of sulfate dry–wet cycle erosion. After 60 cycles, there was less mortar on the specimen’s exterior and around the prism’s edge, making the erosion phenomenon less noticeable. Following 120 cycles, the specimen’s prism cracks on the outside became more severe. The holes and cracks in the mortar on the specimen’s surface steadily enlarged as the number of sulfate dry–wet cycle erosion events increased; the pores were accompanied by noticeable white particles. Preliminary speculation suggests that the precipitated sulfate crystals were erosion products like ettringite or gypsum. The production of gypsum and secondary ettringite, which exert tension in the expanding concrete, is predicted by the attack model put forth by Santhanam et al. [38] in response to a pH shift in the concrete surface. But as soon as the concrete applies compressive force on the solution’s surface, the solution can reach the interior zones that are broken, which causes the cracks to emerge [39].

#### 3.3.2. Mass Loss Rate

**Figure 9:**
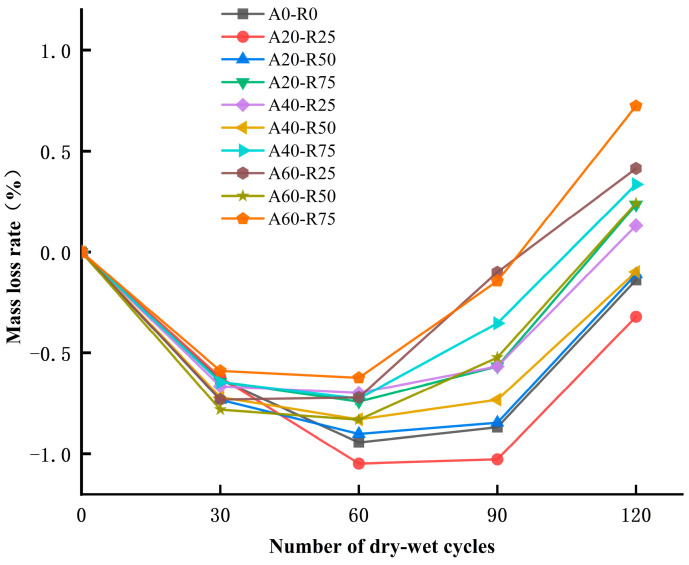
Mass loss rate of ARSCC with different replacement ratios.

The mass loss has been measured in the range of −1.048% to 0.724% for ARSCC mixtures. As seen in Figure 9, in order to highlight the damage, these results are discussed in terms of the mass loss caused to the ARSCC mixtures following exposure to sulfate attacks. As the number of dry–wet cycles increased, the mass loss first reduced and then increased. For instance, the mass loss of the A20-R25 specimen was approximately −1.05% to −0.32%, while the A20-R50 specimen had a strength loss of −0.90% to −0.11%. Compared to combinations comprising other replacement ratios, low replacement ratios of AS and RCA demonstrated a significantly superior resistance to sulfate assault. Furthermore, these findings show that, in comparison to A0-R0 (control), appropriate replacement ratios of AS and RCA result in less mass loss.

#### 3.3.3. Relative Dynamic Elastic Modulus

**Figure 10:**
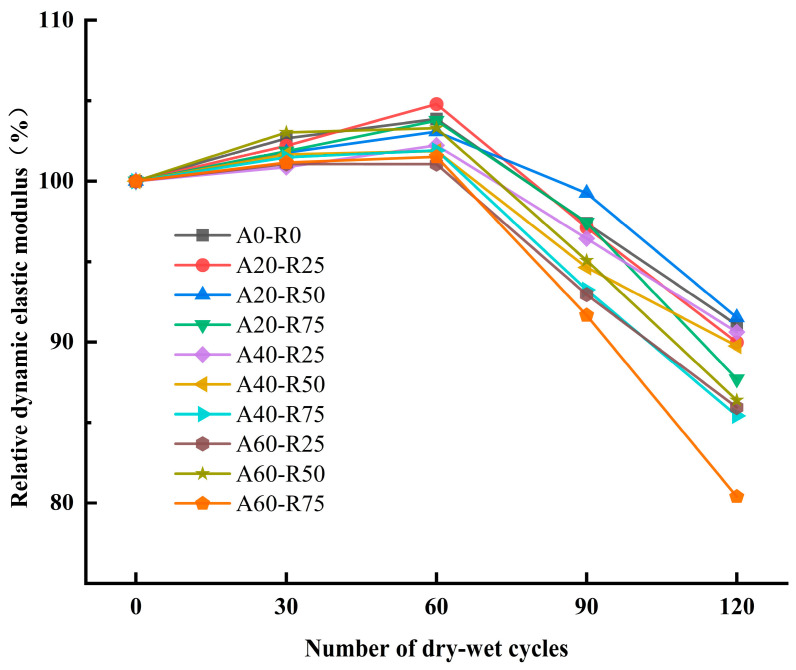
Relative dynamic elastic modulus of ARSCC with different replacement ratios.

The dynamic elastic modulus was used to analyze the mechanical properties of concrete with the effect of freeze–thaw cycles. It assesses the compactness and faults in concrete with ultrasonic velocity [40,41]. For both the freeze–thaw and dry–wet sulfate cycles, the damage mechanisms on the concrete varied, but the damage processes and outcomes were largely the same. Consequently, in this investigation, the damage caused to the ARSCC throughout the sulfate dry–wet cycle can be described by the relative dynamic elastic modulus. Figure 10 illustrates how the relative dynamic elasticity modulus of ARSCC changes at different replacement ratios. All mixture groups showed an increasing tendency for the first sixty cycles, with A20-R25 showing the highest increase at sixty cycles. This is as a result of the low replacement ratios of AS and RCA, which improved cement hydration and made the SCC more compact. However, A20-R50 saw a gradual decline after 60 cycles in contrast to the other groups, including the control group A0-R0. A20-R50 saw a reduction in the relative dynamic elasticity modulus of 11.55%, A0-R0 saw a reduction of 12.80% from 103.87% to 91.07%, and A20-R25 saw a reduction of 14.80% from 104.79% to 89.99%. A20-R50 demonstrated improved resistance to sulfate attacks after higher numbers of cycles. It is possible that the RCA has been fully hydrated after a certain number of cycles and is finding it challenging to react with SO_4_^2−^.

#### 3.3.4. Corrosion Resistance Factor

Table 9 displays the outcomes of the compressive strength following sulfur attacks. All groups’ compressive strengths increased during the first thirty cycles: A20-R50 and A20-R25 increased by 1.9 and 1.7 MPa, respectively, while the control group, A0-R0, increased by 1.5 MPa.

**Figure 11:**
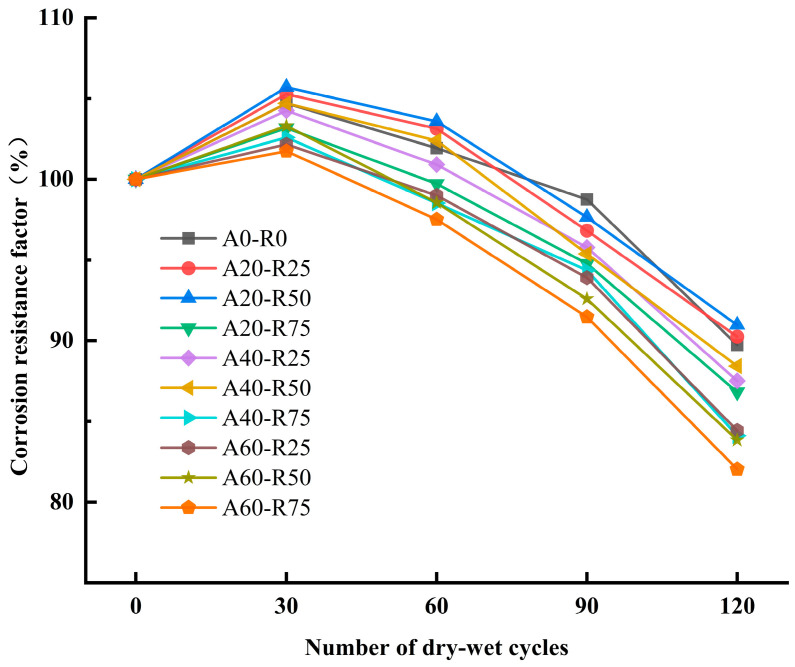
Corrosion resistance factor of ARSCC with different replacement ratios.

The compressive strengths of A20-R50 and A20-R25 declined by 4.9 MPa and 3.1 MPa after 30 to 120 cycles, respectively, compared with their strengths after 30 cycles. The compressive strength of A0-R0 decreased by 4.9 MPa. The findings of the ARSCC’s corrosion resistance factors are displayed in Figure 11. When comparing the corrosion resistance of ARSCC to its compressive strength, the corrosion resistance factor can provide a more accurate picture.

The production of C-S-H gels and the continual hydration of cement could be the cause of the 30-cycle corrosion resistance factor loss in the A0-R0 samples (−4.56%), A20-R25, and A20-R50 samples (−5.28% and −5.71%); however, the microcrack accumulation was enormous [42]. The corrosion products that compressed and condensed during the first thirty cycles showed an increase in compressive strength in ARSCC. Ettringite expansion products may cause volume expansion and expansive stress after 30 cycles, surpassing the maximum tensile strength of ARSCC. This can cause damage and microcracks to occur, hastening the degradation of ARSCC [43]. A20-R50 exhibits improvements in compressive strength and corrosion resistance factor, while A20-R25 is comparable to SCC (A0-R0) in terms of sulfate resistance.

### 3.4. Impermeability Grade and Durability

**Figure 12:**
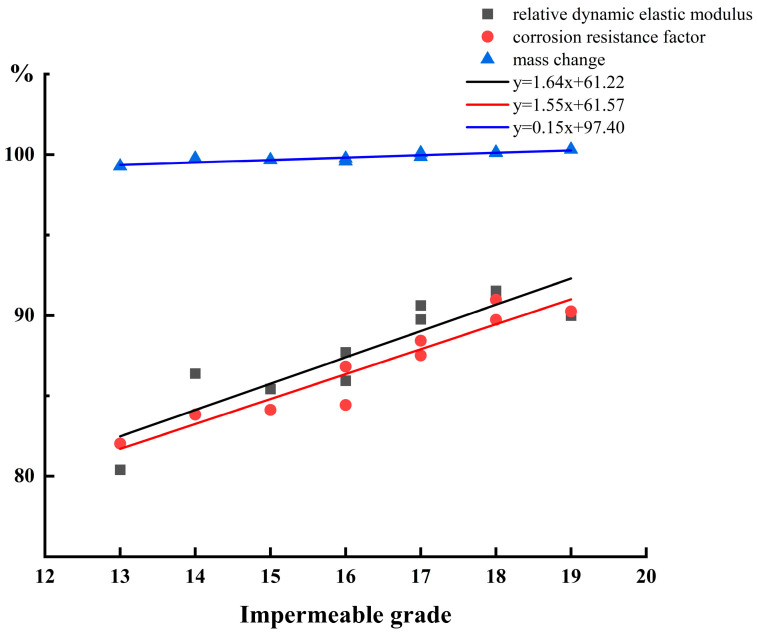
The relationship between impermeability and durability.

Figure 12 illustrates the relationship between durability and impermeability grade. Following linear fitting, it was possible to determine that there are distinct linear relationships, with R^2^ = 0.827 for mass change, R^2^ = 0.803 for relative dynamic elastic modulus, and R^2^ = 0.904 for corrosion resistance factor. It is evident that impermeability grade and durability are positively correlated. This is because water finds it difficult to pass through ARSCC at standard pressures. It is difficult to penetrate into ARSCC with SO_4_^2−^ ions, which also represents the increase in the compactness of ARSCC. Internal pore space is limited, and only the surface of the pore solution reacts. Concurrently, the fitted curve of the relative dynamic elastic modulus not only has the maximum R^2^, but also the maximum slope, demonstrating the benefit of the improved compaction, impermeability, and durability of ARSCC.

## 4. XRD and MIP Analysis

### 4.1. XRD Analysis

**Figure 13:**
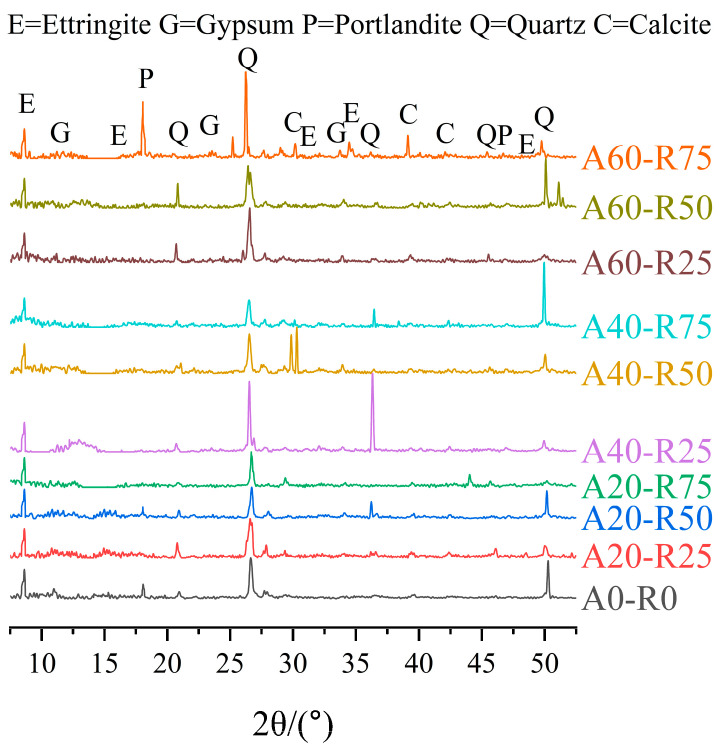
XRD of ARSCC with different replacement ratios after 120 cycles.

The XRD patterns for samples of every mixture following the sulfate attack are seen in Figure 13. The samples of the mixtures A0-R0 and A20-R50 following 120 dry–wet cycles have noticeable ettringite peaks and less intense peaks for Ca(OH)_2_ when compared to samples with varying replacement ratios. Meanwhile, there are clear Ca(OH)_2_ peaks in the A60-R75 sample following 120 dry–wet cycles. Ettringite production is accelerated by corrosion time. As per the earlier research, the process of concrete corrosion due to sulfate attacks can be explained as follows: in the pore solution, the dissolved hydrated calcium hydroxide is devoured by the sulfate ions in the chemical reaction, leading to the creation of gypsum [44,45]. Then, as a result of the cement’s hydration, mono-sulfoaluminate (AFm) or ettringite (Aft) may further form in the micropores. However, AFm may also react with gypsum and pass through into the more stable phase of ettringite, as demonstrated by the chemical reaction represented by Equations (4)–(6) [43]. It is discovered that a high AS and RCA replacement ratio creates more room for products to form and makes it easier for SCC to disrupt the pore structure, which results in the appearance of more severe ettringite peaks [46]. The findings of the mass change and relative dynamic elastic modulus variation in Section 3.3.2 and Section 3.3.3 are supported by the XRD pattern results.

(4) $${Ca\left(OH\right)}_{2}+{Na}_{2}{SO}_{4}+2H_{2}O\to {CaSO}_{4}∙2H_{2}O\left(Gypsum\right)+2NaOH$$

(5) $$\begin{array}{c} 3CaO∙{Al}_{2}O_{3}+3\left({CaSO}_{4}∙2H_{2}O\right)+26H_{2}O\\ \to 3CaO∙{Al}_{2}O_{3}∙3{CaSO}_{4}∙32H_{2}O\left(Ettringite\right)\; \end{array}$$

(6) $$\begin{array}{c} 2\left({CaSO}_{4}∙2H_{2}O\right)+3CaO∙{Al}_{2}O_{3}∙{CaSO}_{4}∙12H_{2}O\left(AFm\right)+16H_{2}O\\ \to 3CaO∙{Al}_{2}O_{3}∙3{CaSO}_{4}∙32H_{2}O\left(Ettringite\right)\# \end{array}$$

### 4.2. MIP Analysis

**Figure 14:**
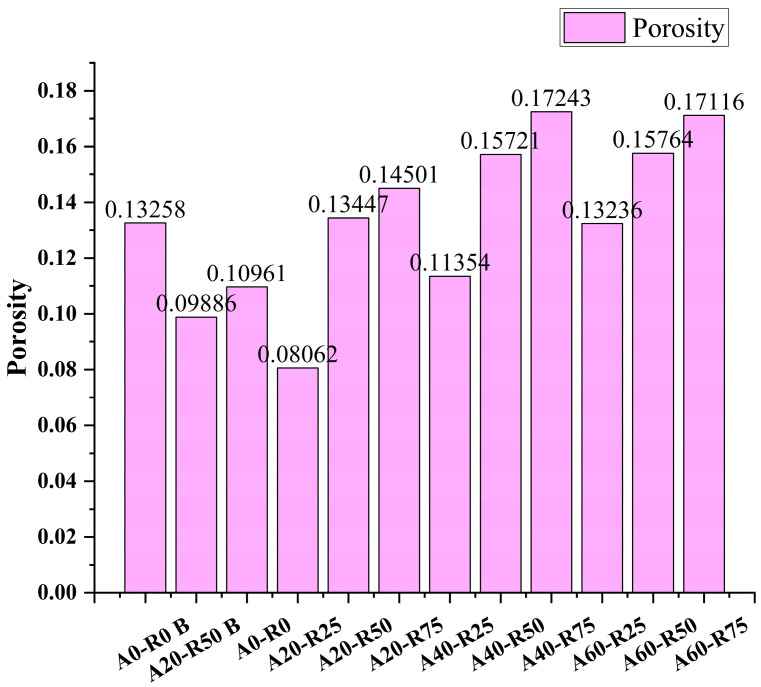
Porosity of ARSCC with different replacement ratios before and after sulfate attack.

**Figure 15:**
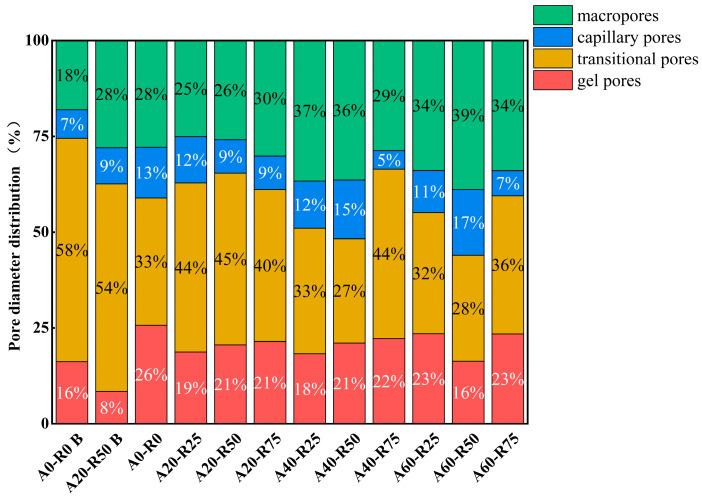
MIP of ARSCC with different replacement ratios before and after sulfate attack.

As illustrated in Figure 14 and Figure 15, MIP tests were performed on samples of the A0-R0 and A20-R50 mixtures prior to the sulfate assault, on A0-R0B and A20-R50B, and on all mixtures following the sulfate attack. The prior work identified four main categories for concrete pores: macropores (width > 1000 nm), capillary holes (diameter 100–1000 nm), gel pores (diameter < 10 nm), and transitional pores (diameter 10–100 nm) [47,48]. In general, it is believed that gel holes may have a significant impact on the durability of concrete; pores bigger than 10 μm may have an impact on the compressive strength of concrete [47]. When the pore distributions of A0-R0B and A20-R50B were compared, it was discovered that A20-R50 had more gel pores and macropores prior to sulfate assault. Following a sulfate assault, the pore distribution of A20-R50 and A20-R25 is comparable to that of A0-R0, but there are fewer gel pores and macropores. This indicates that the corrosion resistance of ARSCC is caused by a proportionate decrease in gel pores and macropores. The mass change and relative dynamic elastic modulus variation results in Section 3.3.2 and Section 3.3.3 are supported by the MIP results.

## 5. Conclusions

This study used the impermeability grade, electric flux, mass loss rate, relative dynamic elastic modulus, and corrosion resistance factor to examine the durability and impermeability of aeolian sand–recycled aggregate self-compacting concrete (ARSCC). The ARSCC was examined under a microscope both before and after the sulfate attack. The study’s primary conclusions are as follows:

1. As the replacement ratios of AS and RCA increase, the compressive strengths of 7 and 28 days first rise and then decrease thereafter, peaking at 20% AS replacement and 50% RCA replacement. This result implies that self-compacting concrete’s compressive strength can be raised by appropriately replacing the amount of AS and RCA.
2. Self-compacting concrete responds to the addition of AS and RCA with exceptional impermeability and durability. The link between durability and impermeability is unambiguously linear. The combination of increasing amounts of AS and RCA lowers the grade of impermeability significantly and speeds up the pace of degradation at later stages because the fine particles of AS and high porosity of RCA create additional channels for water and SO_4_^2−^ erosion, followed by the gradual enlargement of gel pores and macropores.
3. The XRD analysis reveals that ettringite, AFt, and CaSO_4_ are the results of sulfate erosion. A20-R50 has superior compactness and resistance to sulfate assault, as seen by the pore distribution following the sulfate attack. Incorporating AS and RCA increases cement hydration and decreases erosion product generation. The erosion products gradually increase, the pores enlarge, and the resistance to sulfate attack decreases as the AS and RCA replacement ratio increases.
4. A thorough examination of the workability, compressive strength, impermeability grade, electric flux, and resistance to sulfate attack indicates that the appropriate replacement ratio is 20% AS to 50% RCA. This study’s narrow scope prevents a thorough evaluation of ARSCC’s performance; instead, it merely serves as a guide for the usage of ARSCC in specific contexts. It is hoped that increasingly complicated operating conditions, like loaded sulfate coupling, loaded freeze–thaw coupling, and chloride salt freeze–thaw coupling, can be researched for ARSCC.

## Figures and Tables

**Table 1:** Basic properties of cement.

Cement Stability	Ignition Loss Rate (%)	Specific Surface Area (m^2^/kg)	Setting Time (min)		Compressive Strength (MPa)	
			Initial	Final	3 Days	28 Days
Qualified	2.9	354	165	220	27.8	46.5

**Table 2:** Fly ash performance parameters.

Class	Fineness	Ignition Loss Rate (%)	Water Demand Ratio (%)	Moisture Content (%)
II	24.6	4.9	98	0.4

**Table 3:** Basic properties of sand.

Type	Technical Indicators			
	Source	Fineness Modulus	Soil Clay Content (%)	Apparent Density (kg/m^3^)
NS	Urumqi sand field	2.93	0.8	2487.5
AS	Gurbantunggut desert	0.72	0.6	2542.4

**Table 4:** Main components of sand.

Type	Chemical Composition of Sand (%)							
	SiO_2_	Al_2_O_3_	CaO	Fe_2_O_3_	Na_2_O	K_2_O	MgO	Others
NS	90.76	4.59	0.11	0.73	0.39	2.16	0.18	1.08
AS	75.09	11.16	3.66	3.13	2.68	2.34	0.96	0.98

**Table 5:** Properties of coarse aggregate.

Type	Water Absorption (%)	Moisture Content (%)	Packing Density (kg/m^3^)	Apparent Density (kg/m^3^)	Elongated Particle (%)	Crush Index (%)	Robustness (%)
NCA	2.36	0.40	1353.00	2687.22	1.58	8.76	1.2
RCA	3.60	0.81	1204.27	2485.25	3.15	10.44	6.8

**Table 6:** Mix design (kg/m^3^).

Mix Code	W/B	Water	Added Water	NCA	RCA	NS	AS	Fly Ash	Cement	Water Reduce (g)
A0-R0	0.33	169.95	0	848	0	706.86	0	259.37	257.71	3332.4
A20-R25	0.33	169.95	13.89	636	196.06	565.49	141.37	259.37	257.71	3332.4
A20-R50	0.33	169.95	27.77	424	392.13	565.49	141.37	259.37	257.71	3332.4
A20-R75	0.33	169.95	35.29	212	588.19	565.49	141.37	259.37	257.71	3332.4
A40-R25	0.33	169.95	13.89	636	199.94	424.12	282.74	259.37	257.71	3332.4
A40-R50	0.33	169.95	27.77	424	399.89	424.12	282.74	259.37	257.71	3332.4
A40-R75	0.33	169.95	35.29	212	588.19	424.12	282.74	259.37	257.71	3332.4
A60-R25	0.33	169.95	13.89	636	199.94	282.74	424.12	259.37	257.71	3332.4
A60-R50	0.33	169.95	27.77	424	399.89	282.74	424.12	259.37	257.71	3332.4
A60-R75	0.33	169.95	35.29	212	588.19	282.74	424.12	259.37	257.71	3332.4

**Table 7:** Test results of workability.

Mixes	Slump Flow (mm)	J-Ring (mm)	Segregation Resistance (%)
A0-R0	656	12	12
A20-R25	640	11	10
A20-R50	625	10	10
A20-R75	589	7	6
A40-R25	633	6	8
A40-R50	607	4	7
A40-R75	572	3	5
A60-R25	584	4	4
A60-R50	567	2	5
A60-R75	554	1	3

**Table 8:** Test results of impermeability grade and electric flux.

Mix	MSP (MPa)	Impermeability Grade	Electric Flux (C)
A0-R0	1.9	18	598.62
A20-R25	2.0	19	588.79
A20-R50	1.9	18	599.65
A20-R75	1.7	16	643.45
A40-R25	1.8	17	616.14
A40-R50	1.8	17	633.59
A40-R75	1.6	15	684.33
A60-R25	1.7	16	571.32
A60-R50	1.5	14	703.62
A60-R75	1.4	13	753.54

Note: MSP is maximum seepage pressure.

**Table 9:** The results of compressive strength (MPa) after sulfate attack.

Mixes	Number of Dry–Wet Cycles				
	0	30	60	90	120
A0-R0	32.9	34.4	33.5	32.5	29.5
A20-R25	32.2	33.9	33.2	31.2	29.1
A20-R50	33.3	35.2	34.5	32.5	30.3
A20-R75	30.2	31.2	30.1	28.6	26.2
A40-R25	31.6	32.9	31.9	30.3	27.7
A40-R50	32.1	33.6	32.9	30.6	28.4
A40-R75	29.7	30.5	29.3	28.0	25.0
A60-R25	29.1	29.7	28.8	27.3	24.6
A60-R50	29.8	30.8	29.4	27.6	25.0
A60-R75	28.1	28.6	27.4	25.7	23.1

## Data Availability

The data presented in this study are available on request from the corresponding author.
